# Orbital Emphysema as a Consequence of Forceful Nose-Blowing: Report of a Case

**DOI:** 10.1155/2019/4383086

**Published:** 2019-06-18

**Authors:** Yukino Ariyoshi, Hiromichi Naito, Tetsuya Yumoto, Atsuyoshi Iida, Hirotsugu Yamamoto, Noritomo Fujisaki, Toshiyuki Aokage, Kohei Tsukahara, Taihei Yamada, Yasuhiro Mandai, Takaaki Osako, Atsunori Nakao

**Affiliations:** Department of Emergency, Critical Care, and Disaster Medicine, Okayama University Graduate School of Medicine, Dentistry, and Pharmaceutical Sciences, Japan

## Abstract

Orbital emphysema occurs when air enters the soft tissue surrounding the orbit. Although orbital blowout fractures are often caused by face trauma, nontraumatic orbital fractures can also occur but have been rarely described. Here, a case of orbital and palpebral emphysema caused by forceful nose-blowing is presented. Examination uncovered gross swelling of the right eye and discernable subcutaneous emphysema. The patient had normal eye movement and visual acuity. Orbital computed tomography (CT) revealed orbital emphysema secondary to an orbit floor fracture into the maxillary sinus, resulting from high intranasal pressure upon blowing her nose. The patient received conservative management with antibiotics and was given instructions not to sneeze or blow her nose. She fully recovered and all her symptoms completely resolved.

## 1. Introduction

Orbital emphysema is a somewhat rare clinical event. A collection of air within the orbits or eyelids is most commonly associated with fracture and trauma of an orbital bone. Although trauma is the most common etiology of orbital emphysema, nontraumatic spontaneous orbital emphysema due to coughing, sneezing, nose blowing, or any type of straining as a result of iatrogenic otolaryngeal and dental procedures and infectious gas-producing microorganisms have been described [1–5].

We describe a patient with orbital emphysema presenting swelling surrounding the left eye several hours after blowing her nose. Most cases of orbital emphysema resolve spontaneously; however, emergency physicians should be aware that early recognition of orbital emphysema is crucial to prevent possible vision-threatening complications if unrecognized [6]. Since orbital emphysema with no prior history of trauma is a very rare condition in the emergency medicine setting, sharing our experience and review of previous reports of this condition may help emergency physicians decide on therapeutic strategies and initiate immediate management.

## 2. Case Report

**Figure 1:**
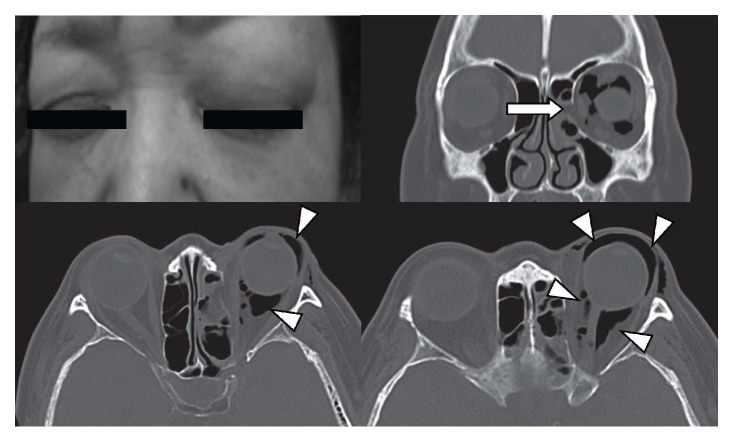
Nontender left periorbital swelling is noted (upper left panel). Computed tomography showed blowout fracture of the left orbital floor with orbital fat herniation (white arrow). Periorbital and orbital emphysema is noted (white arrowhead).

A 59-year-old healthy Japanese female with chronic rhinitis was taken to our emergency department due to a sudden and painless left periorbital swelling following forceful nose-blowing. Examination revealed a gross swelling of the left eye. There was painless palpable emphysema around her left eye; she had normal eyeball movement and visual activity. By ophthalmic consultation, the intraocular pressure was found to be slightly higher in her left eye (20 mmHg) compared to the right (13 mmHg). Noncontrast CT revealed orbital subcutaneous and subconjunctival emphysema and fracture of the median orbital wall of the left eye. Focal herniation of extraconal fat into the ethmoid air cells was noted (Figure 1). Otherwise, her extraocular muscles, optic nerve, and globe were unremarkable. The patient was treated conservatively with prophylactic administration of oral cefdinir and ofloxacin eye ointment and referred to her nearest doctor for outpatient follow-up. The patient was instructed to not blow her nose and advised regarding symptoms requiring immediate review. By the next day, the orbital swelling and periorbital emphysema had partially resolved with normal intraocular pressure.

## 3. Discussion

This case highlights spontaneous orbital emphysema caused by forceful nose-blowing, which may pose a diagnostic challenge. In our patient, orbital emphysema developed when the patient blew her nose by air transfer from the paranasal sinuses into the orbit along a pressure gradient, causing a one-way valve forcing air into the orbit. Orbital fat may function like a ball-valve, becoming displaced from the fracture site during insufflation but falling back to seal the opening under the pressure of trapped air.

Gwaltney et al. measured intranasal pressure during coughing, sneezing, and nose-blowing using fluid dynamic modeling with sinus CT. They reported a mean maximal intranasal pressure of 66 mm Hg during nose-blowing, 4.6 mmHg during sneezing, and 6.6 mmHg during coughing. Intranasal pressures greater than 190 mmHg have been measured during nose blowing with maximal expiratory efforts, offering evidence that air can be introduced into the orbit under substantial pressure [7]. Old trauma to the orbit also might result in fragility of orbital wall and contribute to developing orbital emphysema.

In our patient, chronic inflammation in the maxillary sinus associated with chronic rhinitis may have caused weakening of the orbital floor, making it more susceptible to fracture with high pressure with forceful nose-blowing.

Most orbital emphysema cases require no treatment. However, with progressive emphysema, urgent decompression of the trapped air and acute orbital compartment syndrome is necessary to avoid irreversible vision loss because of mechanical optic nerve stretching or vascular compromise. Extraocular muscle motility and visual acuity are the two most important ophthalmologic functions that should be evaluated emergently in patients with acute orbital trauma. Assessing these abilities may be challenging at times due to head injury severity, level of periorbital soft tissue edema, lack of complete cooperation in alert patients, and reduced consciousness.

Reduced vision after trauma may be caused by intraorbital emphysema, retrobulbar hemorrhage, optic nerve thickening presumably secondary to edema, ruptured globe, detached retina, and optic nerve impingement. These complications are ophthalmic emergencies requiring immediate intervention. Therefore, differentiating benign orbital emphysema from an ophthalmic emergency is critical to avoid adverse sequelae. In nontraumatic cases, the most important differential diagnosis to exclude in acute unilateral eye swelling is orbital cellulitis, which may present with pain on eye movement, visual loss, chemosis, and fever [6].

The diagnosis can be easily made by palpation of the pathognomonic cracking and crepitation on the eyelids. CT plays a crucial role in assessing the intraorbital contents of patients with orbital trauma. Most patients with compromised visual acuity or decreased extraocular muscle motility caused by trauma have abnormalities shown by orbital CT.

Most cases of orbital emphysema resolve spontaneously, and no treatment is needed. Experts generally recommend prophylactic oral antibiotics to treat sinus pathogens for patients with orbital fracture into a sinus [8]. A 23-gauge needle can be used for direct needle drainage for rapid release of pressure and air [9, 10].

In conclusion, clinicians should investigate all suspected orbital blowout fractures with imaging and full ophthalmological examination regardless of trauma history to avoid possible complications like vision loss because of infection and pressure.
